# Use of ceragenins as a potential treatment for urinary tract infections

**DOI:** 10.1186/s12879-019-3994-3

**Published:** 2019-05-02

**Authors:** Urszula Wnorowska, Ewelina Piktel, Bonita Durnaś, Krzysztof Fiedoruk, Paul B. Savage, Robert Bucki

**Affiliations:** 1 Department of Microbiological and Nanobiomedical Engineering, Medical University of Bialystok, Mickiewicza 2c, 15-222, Bialystok, Poland; 2 Department of Microbiology and Immunology, The Faculty of Health Sciences of the Jan Kochanowski University in Kielce, 25-001 Kielce, Poland; 3 Department of Microbiology, Medical University of Bialystok, Bialystok, Poland; 4 Department of Chemistry and Biochemistry, Brigham Young University, Provo, UT 84602 USA

**Keywords:** Urinary tract infection, LL-37 peptide, Ceragenins, Bacterial drug resistance

## Abstract

**Background:**

Urinary tract infections (UTIs) are one of the most common bacterial infections. High recurrence rates and the increasing antibiotic resistance among uropathogens constitute a large social and economic problem in current public health. We assumed that combination of treatment that includes the administration ceragenins (CSAs), will reinforce the effect of antimicrobial LL-37 peptide continuously produced by urinary tract epithelial cells. Such treatment might be an innovative approach to enhance innate antibacterial activity against multidrug-resistant *E. coli*.

**Methods:**

Antibacterial activity measured using killing assays. Biofilm formation was assessed using crystal violet staining. Viability of bacteria and bladder epithelial cells subjected to incubation with tested agents was determined using MTT assays. We investigated the effects of chosen molecules, both alone and in combinations against four clinical strains of *E. coli,* obtained from patients diagnosed with recurrent UTI.

**Results:**

We observed that the LL-37 peptide, whose concentration increases at sites of urinary infection, exerts increased bactericidal effect against *E. coli* when combined with ceragenins CSA-13 and CSA-131.

**Conclusion:**

We suggest that the employment of combination of natural peptide LL-37 with synthetic analogs might be a potential solution to treat urinary tract infections caused by drug-resistant bacteria.

## Background

Urinary tract infections (UTIs) are one of the most common bacterial infections worldwide. There are several risk factors associated with UTI development such as age, pregnancy, sexual intercourse, use of diaphragm and spermicide, delayed post-coital micturition, menopause, catheterization, incontinence, antibiotic use and a previous history of UTI [1]. The main etiological agent of UTIs is uropathogenic *Escherichia coli* (UPEC), which is responsible for more than 80% of all urinary infections [2]. Importantly, most isolates of *E. coli* are multidrug resistant [3]. High recurrence rates and progressively increasing resistance of microorganisms to conventional antibiotics, especially among carbapenem-resistant *Enterobacteriaceae*, makes UTIs a serious social and economic problem [4–6]. It should be noted that some conventional antibiotics used conventionally to fight UTIs, such as fluoroquinolones or trimethoprim/sulphamethoxazole, may no longer be used for empiric treatment due to high resistance rates [7]. Additionally, fluoroquinolones are characterized by significant toxicity, manifested by disorders of the gastrointestinal tract and inflammation of the connective tissue (permanent damage to tendons and cartilage) [8]. Significant drug resistance, which considerably limits the usefulness in the therapy of UTIs, is also reported for doxycycline, a tetracycline antimicrobial characterized by broad spectrum of antibacterial activity, favorable pharmacokinetics and a good safety profile [9]. Furthermore, in the case of chronic and recurrent UTIs infection, the major challenge is eradication of microbial biofilm, which considerably increases bacterial resistance to antibiotics [10].

Among other factors explaining failure of antibiotic therapy of UTIs is ineffective eradication of intracellular pathogens that might avoid antibiotic therapy and host immune response when after uptake are hiding as intracellular microorganism in cytosol of epithelial cells [11, 12]. It is important to note that a majority of currently used antimicrobial agents, such as aminoglycosides and β-lactams, is effective only against extracellular forms of pathogens due to the inability to penetrate and accumulate in the host cells, while others (i.e., macrolides and fluoroquinolones), exhibit poor retention inside host cells [13]. Considering the obstacles in eradication of intracellular pathogens and related with it, recurrence of intracellular bacteria-associated infections, it is suggested that determination of the activity of antibiotics against the intracellular form of the pathogen would more accurate predict the clinical efficacy of agents in in vivo settings. Since intracellular activity of an antibiotic is determined by a variety of factors, from which ability to reach the eukaryotic cell membrane and its subcellular localization, are the most prominent, we hypothesized that membrane-active agents, such as host antibacterial peptides and ceragenins, may demonstrate efficiency in reaching the cytosol.

Substantial efforts have been expended in development of endogenous antimicrobial peptides (AMPs) as new therapeutic options suitable in the treatment of drug-resistant microbial infections. Well studied AMPs include human cathelicidin LL-37, which plays an important role in the first line of mucosal immunity against invading pathogens [14, 15]. In contrast to conventional antibiotics, development of resistance against AMPs is unlikely due to natural origin of AMPs and their mechanism of action. AMPs associate with bacterial membranes, leading to perturbations and loss of permeability barrier function and membrane polarization. Notably, AMPs, as components of innate host defense, are produced by urothelium and renal intercalated cells, both constitutively and in response to microbial assault and are reported to be less toxic than other antibiotics [16, 17]. A recent study by Ibrahim et al. indicated that levels of LL-37 are substantially increased among patients with UTI, both in the patient’s urine, and in the plasma of patients during the period of infection of the urinary tract [18]. In addition, apart from the broad spectrum of antimicrobial activity against bacteria, viruses, fungi and parasites, AMPs have been found to be key modulators of immunity and inflammation, playing critical roles in innate immunity [19–22].

Ceragenins (CSAs), synthetic mimics of endogenous AMPs, are recognized as a novel, effective antibiotic class due to a variety of attractive features, including broad spectrum of killing activity, low synthesis cost, good stability under physiological conditions and a mechanism of action similar to AMPs [23–26]. Studies performed by our research team confirmed that AMP- and ceragenin-induced bacterial killing is rapid [27]. Furthermore, results presented by Pollard et al. indicate that ceragenin CSA-13 retains potent antibacterial activity over the course of 30 serial passages, indicating that there is a small probability that the bacteria will develop resistance to this class of molecules [28]. Given these observations, ceragenins offer a possible effective method for the prevention and treatment of bacterial UTIs. Importantly, results from animal-based studies indicate that ceragenin CSA-13 undergoes urinary excretion, which strongly suggests the possibility of their application in UTIs [29]. Nevertheless, it has not been yet determined if combination therapy of LL-37 peptide with ceragenins can be used as potential treatment against urinary tract infections.

Considering the results described above, we decided to investigate the effects of combinations of LL-37 with selected ceragenins using an in vivo setting of urinary infection: cultures of *E. coli* in planktonic and biofilm form *and E. coli-*infected urinary bladder cells. In this study, we determined the protective effect of LL-37 peptide and ceragenins in relation to epithelium cells of the urinary tract, which can promote survival of uropathogens internalized into the cells and thereby provide the potential to re-infect the host after antibiotic treatment. Considering successful LL-37/ceragenin-mediated eradication of clinical strains of *E. coli* observed during our study, we suggest that therapy with ceragenins will reinforce endogenous LL-37 peptide activity and may be an important option in the treatment of UTIs.

## Methods

### Bacterial strains used in the study

Clinical strains of *E. coli* were obtained from patients diagnosed with urinary tract infection at Independent Public Province Hospital of Jan Sniadecki in Bialystok. The susceptibilities of tested bacterial strains to conventional antibiotics were established by the agar disc diffusion method on MHA agar according to the guidelines of the EUCAST (*ang. The European Committee on Antimicrobial Susceptibility Testing)*. Zones of inhibition were measured to the nearest millimeter and recorded. Susceptibility tests showed that all *E. coli* isolates were resistant to ampicillin, and two of them were resistant to sulfamethoxazolum/trimethoprimum (Table 1).

**Table 1:** Antibiotic sensitivity of clinical strains of *E. coli* (*) used in this study. The antimicrobial inhibition zones were measured and recorded according to CLSI standards. R- resistant; S- sensitive

Antibiotics	E. coli *	E. coli *	E. coli *	E. coli *
Ciprofloksacin	S	S	S	S
Sulfamethoxazolum/Trimethoprimum	R	R	S	S
Norfloxacin	S	S	S	S
Gentamicin	S	S	S	S
Fosfomycin	S	S	S	S
Meropenem	S	S	S	S
Amoxicillin/clavulanic acid	S	R	S	S
Cefepime	S	S	S	S
Cefotaxime	S	S	S	S
Ampicillin	R	R	R	R

### Materials

LL-37 peptide was purchased from Lipopharm.pl (Zblewo, Poland), and the purity of LL-37 was > 98% (as determined by high-performance liquid chromatography [HPLC]). The ceragenins, CSA-13 and CSA-131, were synthesized as described previously [30]. Conventional antibiotics (sulfamethoxazolum/trimethoprimum, ciprofloxacin, norfloxacin, gentamicin, fosfomycin, meropenem, amoxicillin/clavulanic acid, cefepime, cefotaxime, ampicillin and doxycyclinum) were purchased from OXOID (England) and Polfa Tarchomin (Poland).

### Cell culture

Human bladder epithelial cell line T24 (ATCC® HTB­4™), purchased from American Type Culture Collection (ATCC), was cultured at 37 °C with 5% CO_2_ in McCoy’s 5A medium (ATCC, catalog no. 30–2007) supplemented with 10% fetal bovine serum (FBS, ATCC, catalog no. 30–2020). All cell culture-based experiments, with the exception of MTT assays, were performed in serum-free conditions. For this purpose, FBS-containing growth medium was replaced with serum-free medium 4 h before the addition of tested agents at indicated concentrations.

### Antibacterial testing

Minimal inhibitory concentrations (MICs) and minimal bactericidal concentrations (MBCs) of tested compounds were determined using bacteria at the logarithmic phase of growth. Antibacterial agents: LL-37, CSA-13, and CSA-131 and combination of LL-37 with doxycycline (DOX) and ceragenins were tested against four clinical strains of *E. coli* (~ 10^5^ CFU/mL). The MIC and MBC values were determined in Luria-Bertani broth (LB) using the microdilution method described by the reference Clinical and Laboratory Standards Institute (CLSI). The MIC values were determined versus bacterial concentrations of ~ 10^5^ CFU/mL, and the MBC was performed by plating each sample on LB agar.

### Extracellular activities of tested antimicrobial agents

The killing activity of LL-37, doxycycline, ceragenins CSA-13 and CSA-131 and combinations of LL-37 with doxycycline, CSA-13 and CSA-131 were determined against four *E. coli* isolates. Individual colonies of bacteria were diluted to 10^5^ CFU/ml with sterile phosphate-buffered saline (PBS, pH 7.0). The assays were run with different concentrations of LL-37, doxycycline, CSA-13 and CSA-131 (ranging from 2 μM to 50 μM) individually, and combinations of LL-37 with doxycycline, LL-37 with CSA-13 and LL-37 with CSA-131 (ranging from 2 μM to 50 μM; in 1:1 ratio). After 30 min of incubation at 37 °C, samples were diluted 10- to 1000-fold and 10 μl aliquots of each dilution were plated on agar and incubated overnight at 37 °C to determine the number of viable colonies. The colony-forming units (CFU/ml) of the individual samples were determined from the dilution factor. Presented data are an average from three individual experiments. To confirm the killing activity of tested agents, detection of metabolic activity in *E. coli in* suspensions upon treatment with different concentrations of tested compounds (2–50 μM) and their combinations (1:1 ratio) was assessed. After incubation of bacteria with tested compounds (at 37 °C for 1 h), 3-(4,5-dimethylthiazol-2-yl)-2,5-diphenyltetrazoliumbromide (MTT reagent, Sigma Aldrich, USA) at final concentration of 0.5 mg/mL was added to each well, and the 96-well plates were incubated at 37 °C for 1–2 h in the dark. Then, 100 μl of dimethyl sulfoxide (DMSO) was added to each well and the plate was left for 1 h at room temperature to allow the color to develop. The optical density was measured at 550 nm using Varioskan LUX (Thermo Fisher Scientific, Waltham, MA, USA).

### Antibiofilm activities

Formation of biofilm by UTI-associated clinical isolates of *E. coli* in the presence of different concentrations (2–50 μM) of tested compounds and their combinations (1:1 ratio) was assessed using crystal violet (CV) staining (0.1%) in 96-well polystyrene microtiter plates. After incubation of bacterial samples with indicated agents (37 °C for 48 h), the plates were washed with PBS to remove unattached bacteria and stained using 0.1% (*w*/*v*) crystal violet for 15 min at room temperature. Crystal violet was removed, solubilized in 95% ethanol and plates were scanned at 570 nm using Labsystem Varioscan Lux (Thermo Fisher Scientific, Waltham, MA, USA) to determine the optical density of the dye attached to stained biofilms.

### Cytotoxicity assay

The cytotoxic effect of tested compounds and their combinations (1:1 ratio) against bladder epithelial cells was evaluated using MTT assay. For this purpose, T24 (ATCC® HTB­4™) cells were seeded into each well of a 96-well flat-bottom microtiter plate (Sarstedt, Newton, NC, USA) to adhere overnight. After 24 h from seeding, cells were treated with LL-37, doxycycline, CSA-13 and CSA-131 (2, 4, 10, 20 and 50 μM) and combinations of LL-37 with doxycycline, LL-37 with CSA-13 and LL-37 with CSA-131 (ranging from 2 μM to 50 μM; in 1:1 ratio). After 1 h incubation of cells with indicated therapeutic agents (37 °C with 5% CO_2_), the MTT salt working solution (100 μL/well at the final concentration of 0.5 mg/mL) was added to each well and incubated for another 2–4 h. Working medium was removed and DMSO was added (100 μL/well) to solubilize formazan crystals. Absorbance was measured at 550 nm using Varioskan LUX. The percentage of cell viability was calculated as (absorbance of treated cells/absorbance of untreated cells) × 100%.

### Activities of antimicrobial agents against intracellular microorganism

To assess the activity of tested compounds against intracellular pathogens, T24 (ATCC® HTB­4™) cells were seeded at the density of 10^5^ cells/well in a 24-well flat-bottom microtiter plate (Sarstedt, Newton, NC, USA) and cultured at 37 °C with 5% CO_2_ for 24 h to form a confluent monolayer. After this time, medium was removed and wells were washed twice with PBS. Clinical isolates of *E. coli* at log-phase (1 mL, 2 × 10^8^ CFU/mL) were then added to each well and incubated for 2 h at 37 °C. Cells were washed three times with PBS, 5 μg/mL of gentamycin was added to each well to eliminate extracellular *E. coli* and plates were left for further incubation for 2 h. Control wells were washed twice with PBS and T24 (ATCC® HTB­4™) cells were lysed with 0.1% Triton X-100 in PBS for 10 min at 37 °C and plated on agar plates overnight. Subsequently, different molar concentrations (2–50 μM) of LL-37, doxycycline, CSA-13 and CSA-131 and combinations of LL-37 with doxycycline/CSA-13/CSA-131 were added to the remaining wells for 2 h at 37 °C. After incubation, cells were washed three times with PBS and lysed with 0.1% Triton X-100 for 10 min at 37 °C. To determine the viability of intracellular *E. coli* upon treatment with tested agents, obtained samples were diluted 10- to 1000-fold and ten-microliter aliquots of each dilution were spotted on agar plates for ~ 18 h at 37 °C. The CFU (CFU/ml) of the individual samples of intracellular *E. coli* were determined from the dilution factor and were used to calculate the percentage of bacterial outgrowth.

## Results

### Susceptibility of clinical isolates of *E. coli* to conventional antibiotics

The antibacterial activities of conventional antibiotics against clinical strains of *E.coli* obtained from patients diagnosed with UTI were assessed using a disc diffusion assay and are shown in Table 1.

We found that 50% of the *E. coli* isolates were resistant to sulfamethoxazolum / trimethoprimum, which is well-established broad spectrum antibiotic combination with potent bactericidal activity against clinically pathogens causing UTIs. In addition, 100% of *E. coli* strains were resistant to ampicillin. *E.coli* strains employed in this study were sensitive to other conventional antibiotics checked in our experimental settings such as meropenem.

### Antimicrobial activity of LL-37, DOX and ceragenins agents against extracellular bacteria

**Fig. 1:**
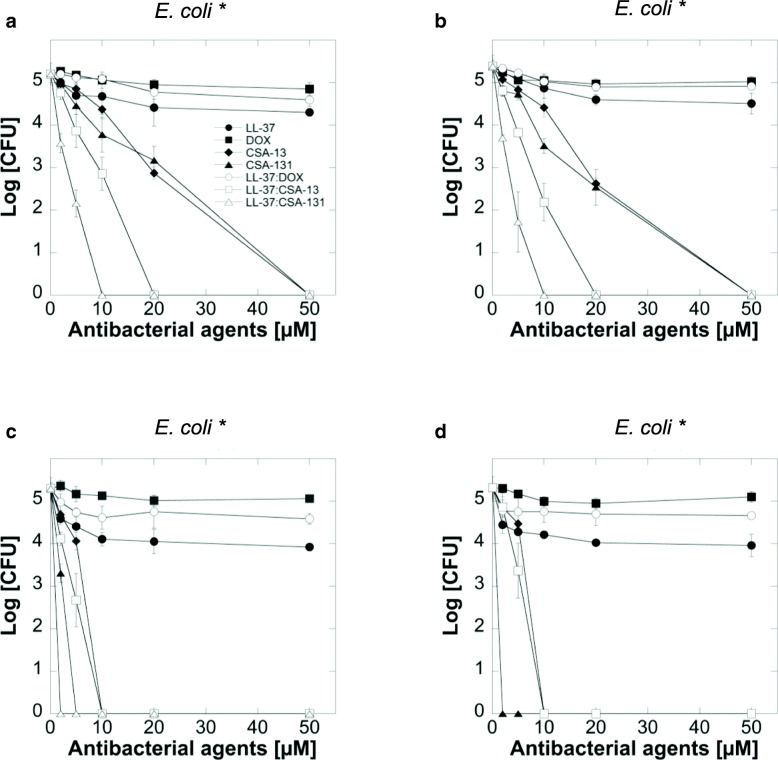
Extracellular antimicrobial activities of LL-37 (filled circles), doxycycline (DOX) (filed squares), CSA-13 (filled diamonds), CSA-131 (filled trianges) and combination of LL-37 with doxycycline (empty circles), LL-37 with CSA-13 (empty squares) and LL-37 with CSA-131 (empty triangles) against four clinical strains of *E. coli* (*) in log phase (Panels **a**-**d**)

To determine the bactericidal activities of LL-37, DOX and ceragenins against extracellular *E.coli* strains, we used a conventional bacterial killing assay (Fig. 1) and MIC/MBC measurements (Table 2).

**Table 2:** MIC and MBC values of LL-37, doxycycline (DOX), CSA-13, CSA-131 and combination of LL-37 with DOX, LL-37 with CSA-13 and LL-37 with CSA-131 against clinical strains of E. coli*

	μg/mL			
	E. coli *	E. coli *	E. coli *	E. coli *
LL-37	32/64	32/64	128/256	128/128
DOX	2/4	4/8	2/16	2/8
CSA-13	2/2	2/2	2/2	2/4
CSA-131	4/8	4/8	4/4	4/16
LL-37:DOX	2/8	4/8	4/8	4/8
LL-37:CSA-13	2/4	4/8	1/2	1/2
L-37:CSA-131	2/2	2/4	2/4	4/8

**Fig. 2:**
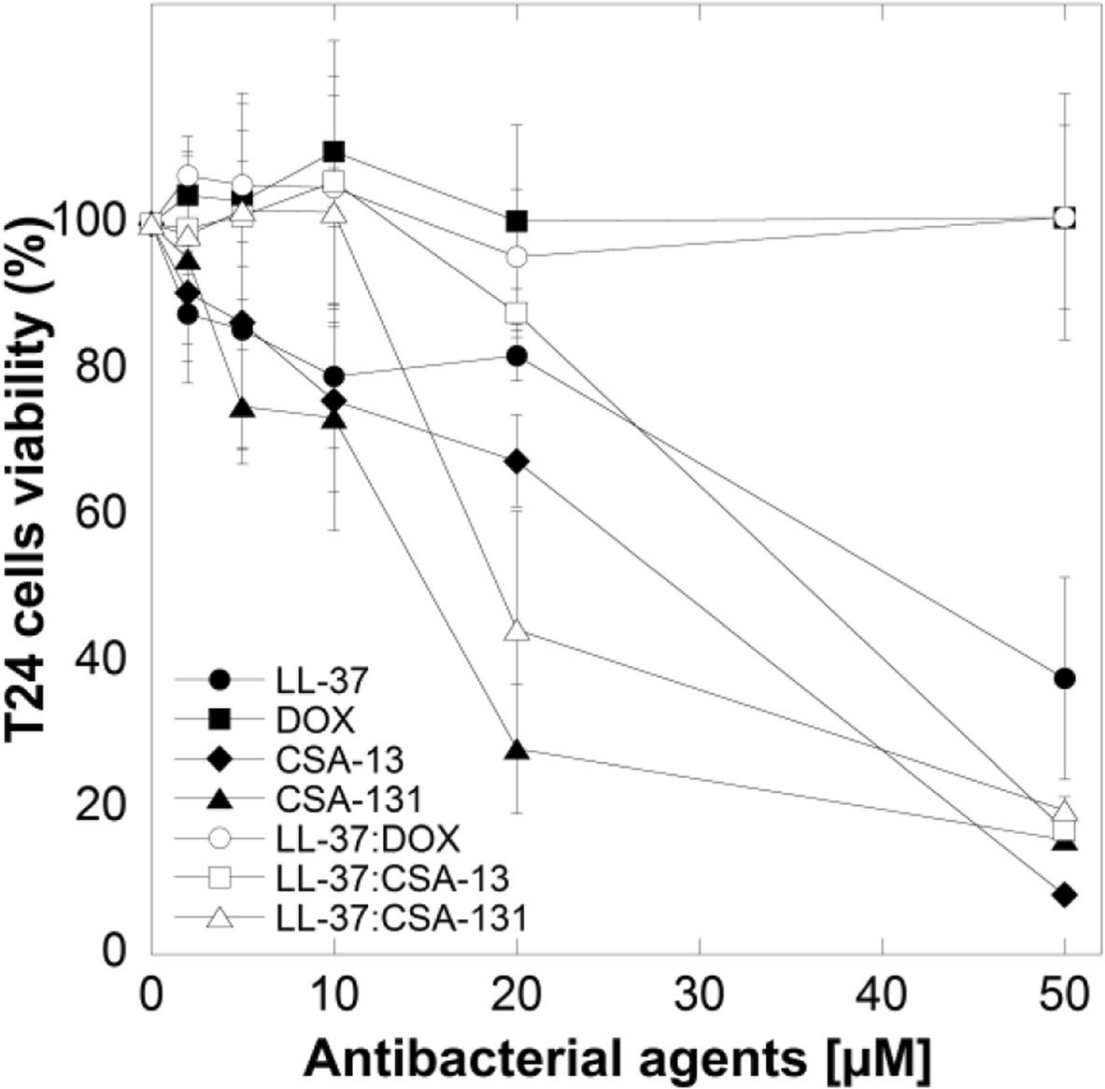
Cell viability of T24 bladder cancer cell line after treatment with LL-37 (filled circles), doxycyclinum (filled squares), CSA-13 (filled diamonds), CSA-131 (filled triangles), LL-37 with doxycyclinum (empty circles), LL-37 with CSA-13 (empty squares) and LL-37 with CSA-131 (empty triangles)

**Fig. 3:**
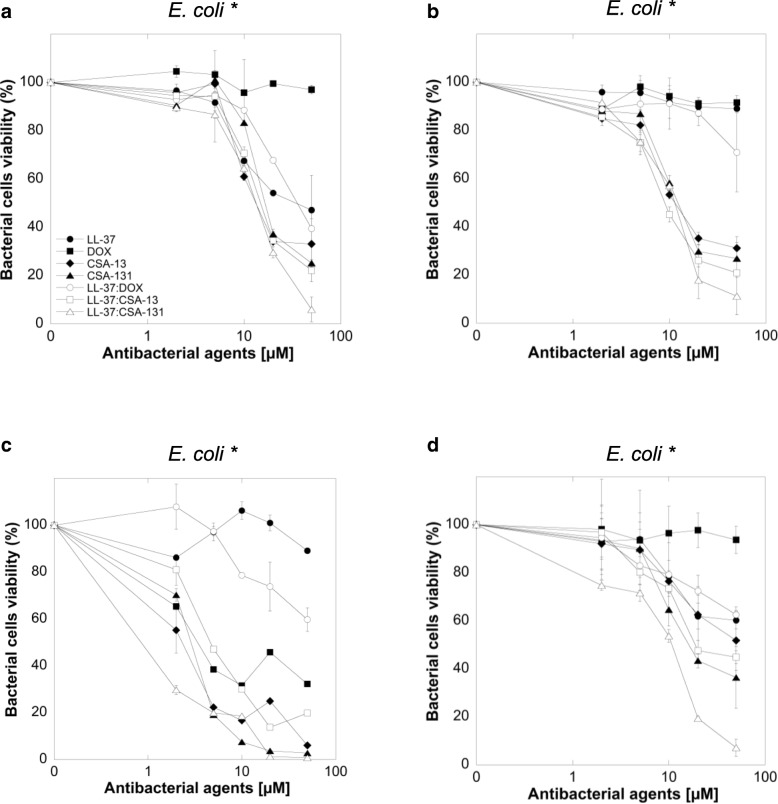
Antibacterial activities of LL-37 (filled circles), doxycyclinum (filled squares), CSA-13 (filled diamonds), CSA-131 (filled triangles) and comibination of LL-37 with doxycyclinum (empty circles), LL-37 with CSA-13 (empty squares) and LL-37 with CSA-131 (empty triangles) against four clinical strains of *E. coli* for 1 h at 37 °C (Panels **a**-**d**). After the addition of MTT salt, cells were further incubated for 1 h at 37 °C

Susceptibility data from our experiments demonstrate that ceragenins (CSA-13, CSA-131) have stronger bactericidal activities against *E. coli* than LL-37 or doxycycline against clinical strains of pathogen. Additionally, when compared to ceragenins alone, the combinations of LL-37 with ceragenins had significantly stronger activities against *E. coli* (Fig. 1a, b, c and d), with the most prominent effect observed for the combination of LL-37 with CSA-131. Importantly, at doses that exert bactericidal effects (i.e. 2–10 μM), low toxicity against mammalian cells was observed (Fig. 2). The MICs and MBCs of LL-37, DOX, ceragenins and combination of LL-37 with DOX and ceragenins against clinical isolates of *E. coli* are shown in Table 2. Overall, the in vitro activities of the combination of LL-37 with CSA-131 (average MIC for all tested strains ~ 2.5 μg/mL) was somewhat greater than CSA-131 (MIC ~ 4 μg/mL) and was much greater than that of LL-37 (MIC ~ 80 μg/mL) both used individually. Similar results were obtained with CSA-13. Doxycycline activity was not increased when combined with LL-37 peptide. To confirm these observations, we assessed the metabolic activity recorded in bacterial samples after 1 h of treatment with different antibacterial agents at varied concentrations. For this purpose, MTT assay, based on the conversion of MTT component to purple, non-soluble formazan precipitate by viable cells with active metabolism, was employed. As expected, the antibacterial activities of ceragenins, but not LL-37 or doxycycline resulted in significant decrease of *E.coli* cell viability (Fig. 3 a-d). The antibacterial activities of ceragenins in combination with LL-37 were strongly higher than with doxycycline. These observations show that combinations of ceragenins with LL-37 are suitable for developing effective treatments against *E. coli* UTIs.

### Biofilm formation in the presence of ceragenins

**Fig. 4:**
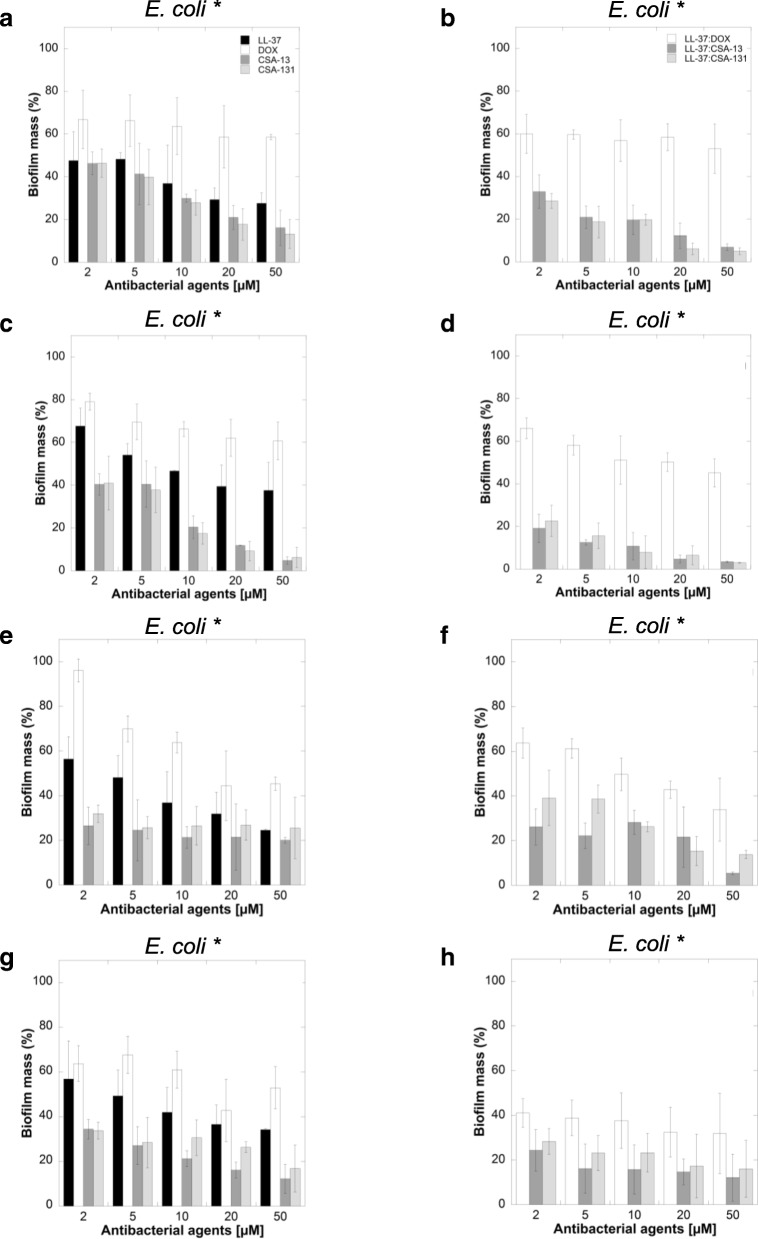
Antibiofilm activities of LL-37 (black columns), doxycycline (DOX) (white columns), CSA-13 (dark grey columns), CSA-131 (light grey columns) against E. coli (*) (Panels **a**, **c**, **e**, **g**) and antibiofilm activities of combination of LL-37 with doxycycline (white columns), LL-37 with CSA-13 (dark grey columns) and LL-37 with CSA-131 (light grey strips) against *E. coli* (*) clinical strains after 48 h (Panels **b**, **d**, **f**, **h**)

Quantification of biofilm mass with the crystal violet assay (Fig. 4) showed that ceragenins alone exert significant activity against *E. coli* and effectively prevent biofilm formation. Moreover, LL-37 in combination with ceragenins had considerable efficacy in preventing biofilm formation by *E. coli* compared to LL-37 with DOX, which were unable to inhibit biofilm formation at tested concentrations. In some cases, more than 50% higher activity of ceragenins in combination with LL-37 at the same molar ratio was observed. Notably, DOX alone showed no antibiofilm properties against *E. coli*.

### Bladder cell viability upon treatment with antimicrobial agents

The results of cell viability assays after the incubation of the T24 cell line with antibacterial agents at different concentrations are displayed in Fig. 2. LL-37 peptide, doxycycline and ceragenins alone and in combination with LL-37, at low concentration (i.e. 1–10 μM), did not significantly affect the survival of T24 cells. Higher doses of these antimicrobial agents caused a dose-dependent cytotoxic response resulting in cells lysis. Nevertheless, strong bactericidal effects were observed at lower, non-cytotoxic doses of tested agents, which highlights their safety in the treatment of *E. coli*-caused UTIs.

### Bactericidal activity of ceragenins combined with LL-37 peptide against intracellular E. coli

**Fig. 5:**
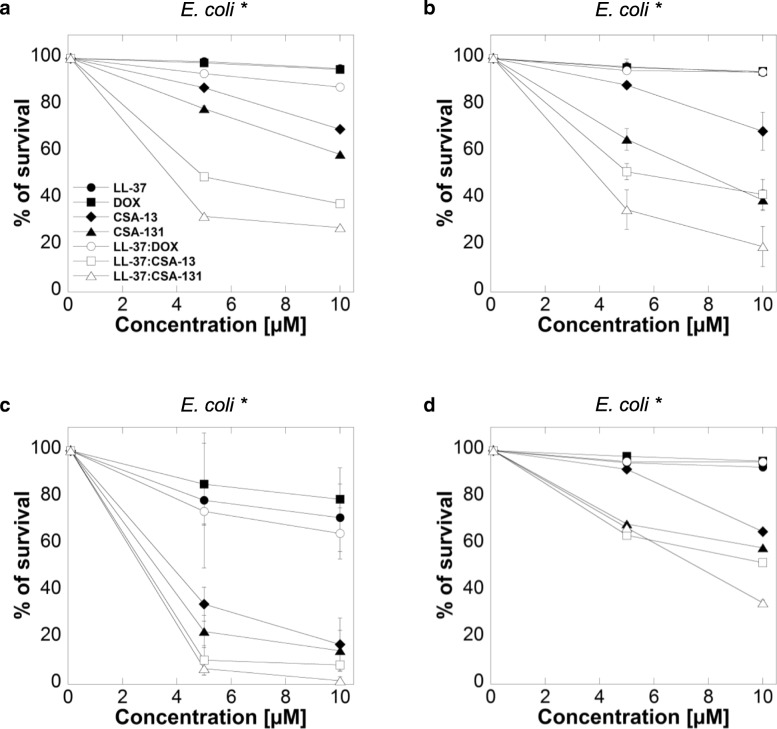
Intracellular killing efficacies of LL-37 (filled circles), doxycyclinum (filled squares), CSA-13 (filled diamonds), CSA-131 (filled triangles) and combination of LL-37 with doxycyclinum (empty circles), LL-37 with CSA-13 (empty squares) and LL-37 with CSA-131 (empty triangles) against four clinical strains of E. coli* within T24 cells (Panels **a**-**d**). The concentrations of used antibacterial agents were 5 and 10 μM; incubation time was 2 h

To investigate whether combination therapies of LL-37 peptide with doxycycline/ceragenins are more effective than the antibacterial compounds alone in an in vitro model mimicking the site of infection, we evaluated the killing effectiveness of antimicrobial agents against intracellular pathogens, i.e. bacterial cells with the ability to escape antibiotic therapy and re-infect the host after the end of treatment. Tested agents were investigated at molar combinations ranging from 5 to 10 μM, since higher doses were toxic against tested cell line (Fig. 2). The combination of LL-37 with ceragenins (CSA-13 and CSA-131) was found to be more effective in eliminating intracellular *E. coli* than LL-37, CSAs or doxycycline alone. Significantly, we showed that combination of LL-37 with CSA-131 killed approximately 65 and 79% of the intracellular *E. coli* at 5 μM and 10 μM concentrations, respectively (Fig. 5). In contrast, at the same concentration antibacterial agents alone killed fewer intracellular organisms: LL-37 killed 8–12%, doxycycline killed 6–20%, CSA-13 killed 29–45% and CSA-131 killed 41–57%, depending on the tested bacterial strain. Importantly, the intracellular bacterial percent killing increased with higher concentration of combination of LL-37 with CSAs (Fig. 5 a-d). Consequently, 10 μM of LL-37 with CSA-131 completely eradicated the intracellular *E. coli* strain (Fig. 5c). The applied ceragenins had an increased bactericidal effect for *E. coli* in combination with the LL-37 peptide, the concentration of which increases at the site during UTIs. Importantly, these results suggest that this combination could prevent the recurrence of infections through elimination of intracellular bacteria.

## Discussion

The urinary tract is protected with numerous defense mechanisms in order to prevent microbial infections. Human antimicrobial peptides are a part of human innate immunity and contribute to the first line of defense against infections [14]. AMPs have been identified as a potent chemoattractant for innate and adaptive immune cells, drawing them to the site of infection or inflammation [3]. Moreover, they are capable of binding and neutralizing lipopolysaccharides (LPS), an activity that promotes angiogenesis and wound healing, and are characterized by anti-tumor activity [31]. It is known that LL-37 is constitutively expressed at low levels in urothelial cells, renal epithelial cells, and neutrophils [32]. Ronald A. showed that reduced secretion of the LL-37 peptide is associated with increased incidence of UTIs [33]. Closely related are results from Borregaard N. et al. showing that microbial assault causes rapid and strong increase in endogenous release of LL-37 peptide from epithelial cells determining the protective role of this compound in urinary tract [34]. Due to their cationic nature and membrane activity, LL-37 and ceragenins are display broad spectrum and selective antimicrobial activity against bacteria, fungi, viruses, and parasites [18, 23, 25, 29]. Importantly, LL-37 and ceragenins shows low toxicity to human cells [35]. Moreover, we have to point out that in this study, we investigated the human bladder epithelial transformed cell line T24 (ATCC® HTB­4™) which are susceptible to AMPs and ceragenins. In agreement with Lehmann J. et al. we also believe that it is very likely that the cytotoxicity of LL-37 peptide and ceragenins would be much less with primary cells [36]. In addition, because ceragenins are not peptide based, they are resistant to digestion by proteases [26].

Currently, numerous novel antibiotics or their combinations aimed to treat urinary tract infections are being tested in clinical trials. For example, combinations of beta-lactam antibiotics with beta-lactamase inhibitors, siderophore antibiotics, novel fluoroquinolones, novel aminoglycosides, and novel tetracyclines are being evaluated for the potential to eradicate bacterial pathogens causing UTIs. Tetracyclines are a class of widely used antibiotics with broad-spectrum activity against both Gram-positive and Gram-negative bacteria, as well as against intracellular organisms [37], and doxycycline, a tetracycline antimicrobial, has been identified as an option for treatment of [9]. Importantly, Tang et al. showed that antimicrobial activity of DOX against *Klebsiella pneumoniae* carbapenemase (KPC) isolates might be additionally enhanced by combining doxycycline with gentamicin/amikacin [38]. Jernigan et al. also showed that combination therapy of doxycycline with gentamicin was more effective against KPC-producing isolates than monotherapy [39]. Nevertheless, to the best of our knowledge, the combinatory effects of doxycycline with LL-37 and synergistic effects of LL-37 with ceragenins against multi-drug resistant *E. coli* responsible for UTIs have been not studied.

An important factor promoting recurrent UTIs is microbial survival inside epithelial cells of the urinary tract. Elimination of these intracellular organisms is a challenge in the development of novel antimicrobial. These intracellular bacteria contribute to episodes of recurrence of UTIs, and within 6 months of the first event recurrence occurs in nearly 50% of cases. Recurrent UTIs typically are not life-threatening, but the high frequency increases health-care costs and has negative impacts on patients’ life quality. Importantly, combination of ceragenins with LL-37 peptide presented in this study shows stronger intracellular activity against uropathogenic *E. coli* than both agents alone, which is in agreement with other studies clearly indicating that combinatory therapy is more efficient in eradication of multi-drug resistant (MDR) bacterial infections when compared with monotherapy [38].

Considering that the mechanism of action of AMPs is based on membrane perturbation and the ability of AMP-derived therapeutic agents to internalize into infected cells, it is not unexpected that AMPs gain access to intracellular pathogens. We have observed that LL-37 combined with its non-peptide mimics, ceragenins CSA-13 and CSA-131, or doxycycline, may reduce the emergence of resistance during antimicrobial therapy and limit the recurrence of infection after the end of treatment. This is the first study to evaluate combined antibiotic therapy with LL-37 and ceragenins against uropathogenic *Escherichia coli* (UPEC). To date, a compelling number of reports demonstrate that combinatory-based therapies result in a synergistic effects that allow dose reductions potentially limiting side effects [40].

Understanding antibiotic susceptibility patterns is essential for treatment of severe *E. coli* infections [41]. Nevertheless, the development of antibiotic-resistant strains has limited the therapeutic options available to physicians. In this study, we observed strong bactericidal activities of ceragenins, CSA-13 and CSA-131, and, although ceragenins alone showed higher activities against *E. coli* than doxycycline or LL-37, the combinations of these compounds with LL-37 seemed to further enhance their bactericidal activity (Fig. 1). Our results showed similar antibacterial effectiveness of combinations of CSA-13/CSA-131 with LL-37 against *E. coli* clinical strains, since they displayed similar MIC values (Table 2). The observation of a greater effectiveness of ceragenins when combined with LL-37 is supported by the previous reports indicating that both LL-37 and ceragenins can bind to the negatively charged microbial outer membrane by electrostatic and hydrophobic actions, leading to bacterial cell death [42].

Studies have highlighted the anti-biofilm activity of AMPs, including LL-37 [43]. de la Fuente-Nunez C. et al. developed the innate defense regulator 1018, DJK-5, and DJK-6, which are synthetic cathelicidin-derived anti-biofilm peptides and are characterized by a broad-spectrum activity against multidrug resistant organisms [44]. Moreover, Anunthawan et al. established that KT2 and RT2, which are the two tryptophan-rich cationic antimicrobial peptides, presented anti-biofilm activity at sub-MIC levels against the MDR, *E. coli* O157:H7 strain. They also showed that these AMPs were able to prevent biofilm formation and eradicate mature biofilms at low concentration. KT2 and RT2 associate with negatively-charged LPS molecules and subsequently interact with cytoplasmic membrane phospholipids [45]. Recently, new novel anti-biofilm peptides have been developed based on a dendrimeric (dimeric) scaffold, such as SB056 [46].

Our results demonstrate that employing two therapeutic agents with membrane-permeabilizing properties strengths the anti-biofilm activities of these compounds. An important feature of UPEC strains is the presence of virulence factors that increase the ability of bacteria to colonize and persist in the urogenital tract [47]. It is generally accepted that *E. coli* forms bacterial communities known as biofilms, primary on urinary catheters, as well as on and within bladder epithelial cells. In biofilm form, UPEC is protected from antimicrobial therapy and the host immune system [48, 49]. Soto S.M. et al. demonstrated that recurrent UTIs have been associated with the capacity to form biofilm in vitro [50]. Published data show that antimicrobial peptides possess a potential for inhibition of biofilm [43]. In our research we observed that LL-37 in combination with ceragenins inhibited the formation of biofilm by ca. 80% at 5 μM. These combination therapies are a promising approach to biofilm-related infections. Single antibiotic administration at high concentrations may cause toxicity and lead to drug-resistant strains. Co-administration of anti-biofilm peptides offers a safety strategy in UTIs.

To date, it was found that biofilm resistance to antimicrobial agents is determined by poor penetration and reduced diffusion of antibiotics into the biofilm, nutrient limitation, the presence of persister cells, slow growth rates or increased horizontal transfer of resistance genes [51, 52]. Peptides with anti-biofilm properties that can kill both Gram-negative and Gram-positive bacteria and also fungi in biofilms or inhibit development of biofilms are promising therapeutics as alternative treatments to conventional antibiotics [43]. Our previous research shows that ceragenins effectively prevent microbial biofilm formation including biofilms comprised of highly resistant strains [24]. Olekson M. et al. showed that ceragenins have a broad spectrum activity against mixed-species biofilms affecting cell viability, cell morphology, and matrix production, which highlights the role of ceragenins as stable, potent antimicrobials [53].

## Conclusions

**Fig. 6:**
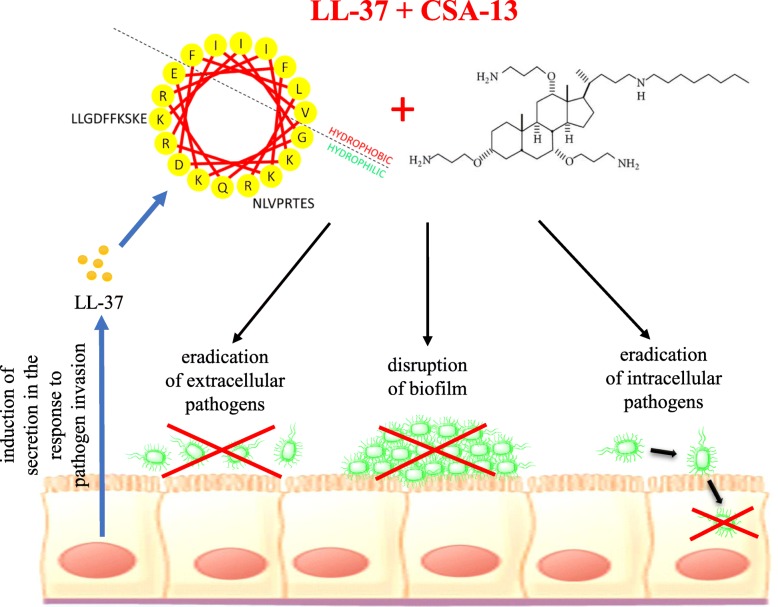
Spectrum of protective effects mediated by LL-37 peptide in combination with ceragenin CSA-13. The figure was prepared using ChemBioDraw Software

These studies demonstrate that combinations of LL-37 with ceragenins possess the potential to be used as novel strategy to treat UTIs caused by antibiotic-resistant *E.coli* strains. Extra- and intracellular activity, and importantly, anti-biofilm activity of these combinations appear well suited for treatment of recurrent UTI (Fig. 6).

## Abbreviations

AMPs: Antimicrobial peptides
ATCC: American Type Culture Collection
CFU: Colony-forming units
CLSI: Clinical and Laboratory Standards Institute
CSAs: Ceragenins
CV: Crystal violet
DMSO: Dimethyl sulfoxide
DOX: Doxycycline
EUCAST: The European Committee on Antimicrobial Susceptibility Testing
FBS: Fetal bovine serum
KPC: *Klebsiella pneumoniae* carbapenemase
LB: Luria-Bertani broth
LPS: Lipopolysaccharides
MBC: Minimal bactericidal concentration
MDR: Multi-drug resistant
MIC: Minimal inhibitory concentrations
PBS: Phosphate-buffered saline
UPEC: Uropathogenic *Escherichia coli*
UTIs: Urinary tract infections
